# Editorial: Chemical Design and Biomedical Applications of Disulfide-rich Peptides: Challenges and Opportunities

**DOI:** 10.3389/fchem.2020.586377

**Published:** 2020-10-23

**Authors:** Diana Imhof, Durba Roy, Fernando Albericio

**Affiliations:** ^1^Pharmaceutical Biochemistry and Bioanalytics, Pharmaceutical Institute, University of Bonn, Bonn, Germany; ^2^Department of Chemistry, Birla Institute of Technology and Science-Pilani, Hyderabad, India; ^3^Peptide Science Laboratory, School of Chemistry and Physics, University of KwaZulu-Natal, Durban, South Africa; ^4^Institute for Advanced Chemistry of Catalonia, Instituto de Quimica Avanzada de Catalunya–Consejo Superior de Investigaciones Cientificas (IQAC-CSIC), Barcelona, Spain; ^5^CIBER-BBN, Networking Centre on Bioengineering, Biomaterials and Nanomedicine, Department of Organic Chemistry, University of Barcelona, Barcelona, Spain

**Keywords:** disulfide (SS) bridges, peptide drug, biomedical application, NMR, chemical design

The last two decades have witnessed of a revolution within the peptide field. From being considered just biochemical tools to becoming a real alternative to both small molecules and biologics as drugs. Currently, there are more than 90 peptides in the market ranging from small di- or tripeptides to large peptides containing more than 30 and even 40 amino acids (Henninot et al., 2018; Al Shaer et al., 2020). Among the last arrivals into the market, ixazomib (Ninlar®), which is an N-acylated, C-boronic acid dipeptide approved by the Food and Drug Administration (FDA) in 2015 for the treatment of multiple myeloma, and macimorelin (Macrilen®), a small pseudopeptide formed by three residues, approved by FDA in 2016 for the treatment of adult growth hormone deficiency, are examples of the first group (Al Shaer et al., 2020). Adlyxin (lixisenatide®), a 44 amino acid peptide, authorized by the same agency in 2016 for the treatment of the diabetes, could exemplify the second group (Al Shaer et al., 2020).

Fifty years ago, it was entirely predictable that small peptides entered into the market. The dipeptide enapril, for example, which is still one of the most prescribed medications around the world, was marketed in 1984. The reason is that they were synthesized as if they were small molecules. The possibility of having a sufficient amount and, more importantly, the required purity for human administration was unthinkable for medium-sized peptides. In those days, the future of this kind of peptides was more as biochemical tools than as drugs.

What was the impetus that changed this paradigm? Without a doubt, this is the development of the solid phase peptide synthesis (SPPS) technology by Merrifield (1963), and most importantly, its subsequent refinement. SPPS is a clear example of how transformative research can be done from basic research. In 1963, Merrifield published a new and a very efficient method for the synthesis of a simple tetrapeptide (Leu-Ala-Gly-Val) (Merrifield, 1963). At first, the method was not well-received by the entire Academy, it was even criticized. Thus, a reviewer of the first article characterizes SPPS as a “travesty, …not chemistry at all, a concept which should be suppressed by the community” (Marshall, 2008). However, the boom of peptide-based drugs is due to the implementation of SPPS in both research and production modes. The use of SPPS in research allows the synthesis of small amounts of peptides in a very short period of time and with sufficient purity that after a simple purification step they can be screened in front of a biological target, accelerating enormously the drug discovery process. Furthermore, the SPPS applied to peptide production allows the preparation of large peptides in a multi-kg scale with the same purity standards to those applied to small molecules.

In a very simple manner, SPPS can be interpreted as the use of a polymeric solid protecting group (resin) for the C-carboxyl function of the C-terminal amino acid, which usually stands in the form of ester (acid peptides) or of amide (amide peptides) (Behrendt et al., 2016). Thus, the amino acid residues are incorporated with the Nα-amino and the side-chain functions are protected. The first one with a temporary protecting group fluorenylmethoxycarbonyl (Fmoc), which is removed in each step, and, mainly, a tert-butyl (tBu) or trityl (Trt) group for the side-chain, which are removed in the last global deprotection and cleavage step (Behrendt et al., 2016). The use of the resin (polymeric protecting group) enables efficient work-ups, such as filtration and washings, allowing the use of large excesses of reagents (Fmoc-protected amino acids, coupling reagents, piperidine solution to remove the Fmoc) to force quantitative yields assuring excellent purities of the target peptides. SPPS can even facilitate some chemistries when compared with solution mode, such as cyclization, due to the pseudo-dilution phenomenon inherently associated with the chemistry on the solid phase (Behrendt et al., 2016).

From a structural point of view, a great majority of all natural and synthetic peptides contains two bonds, the amide/peptide and the disulfide bond. Whereas, the peptide bond forms the basis of the primary structure of a peptide/protein, the disulfide bridges are key for determining the secondary and tertiary structure of peptides and proteins. Furthermore, they are responsible for the integrity of the multi-subunit peptides/proteins, such as those occurring in insulin or in antibodies.

The *peptide as drugs* journey started with a disulfide-containing peptide, when Vicent du Vigneaud, laureate of the Nobel Prize in Chemistry in 1955, performed the first synthesis of a peptidic hormone, namely oxytocin (Du Vigneaud et al., 1953). The neuropeptide oxytocin is a nine amino acid peptide containing a disulfide bridge and nowadays has a multi-kg-scale yearly production.

Table 1 lists the Cys-containing peptides approved by the FDA and European Medicines Agency (EMA) as drugs during the last 20 years. In addition, both agencies have approved a large number of antibodies and proteins, which also contain Cys and disulfide bridges.

**Table 1:** List by alphabetical order of Cys-containing peptide-based drugs approved by FDA and EMA in the last years (Henninot et al., 2018; Al Shaer et al., 2020).

**Active ingredient**	**Trade name**	**Medical indication**	**Structural characteristics**
Atosiban	Tractocile™	Tocolytic, labor suppressant	One disulfide bridge between a Cys and β-mercaptopropionyl in a nine residue peptide
Carbetocin	Pabal™	Minimize bleeding after cesarean	One thiotether bridge between a Cys and γ-butanoyl in a nine residue peptide
Etelcalcetide	Parsabiv™	Calcimimetic, hyperparathyroidism in patients under hemodialysis	Intermolecular disulfide bridge between a D-Cys containing peptide (7 D-amino acids) and L-Cys-OH
^68^Ga-DOTA-TOC		Scintigraphic imaging	^68^Ga chelated by DOTA that is attached to [Tyr3]-octreotide (one disulfide bridge in an eight residue peptide)
Insulin degludec	Tresiba™	Diabetes	Two chains (60 amino acids) with two intra- and one inter-chain disulfide bridges and a hexadecanedioyl γ-L-glutamyl is bounded to Lys side-chain
Linaclotide	Linzess™	Irritable bowel syndrome with constipation	Three disulfide bridges in a 14 amino acid peptide
^177^Lu-DOTA-TATE	Lutathera™	Theranostic for gastroenteropancreatic neuroendocrine tumors	^177^Lu chelated by DOTA that is attached to [Tyr3]-octreotate (one disulfide bridge in an eight residue peptide)
Nesiritide	Natrecor™	Cardiovascular fluid homeostasis	One disulfide bridge in a 32 amino acid peptide
Pramlintide	Symlin™	Diabetes	One disulfide bridge in a 37 amino acid peptide
Peginesatide	Omontys™	Erythropoietic	Two copies of a 20 amino acid peptide with one disulfide bridge linked to a (Lys)_3_ core, which also holds two PEG (20.000) chains
Plecanatide	Trulance™	Chronic idiophatic constipation	Two disulfide bridges in a 16 amino acid peptide
Ziconotide	Prialt™	Chronic pain	Three disulfide bridges in a 25 amino acid peptide

This special issue of Frontiers entitled “Chemical Design and Biomedical Applications of Disulfide-rich Peptides: Challenges and Opportunities” illustrates through six excellent contributions what is the state of the art in this field. One major challenge in the development of disulfide-rich peptides as novel drugs is the improvement and easier access to synthetic material, which can be analytically characterized in a manner that is conclusive. A suitable example, highlighting the complexity of the production and the tandem mass spectrometry analysis of a peptide toxin that contains an inhibitory cysteine knot (ICK) motif, is provided by McCarthy et al. This report demonstrates the existence of misfolded peptides with non-native single disulfide bonds of the peptide toxin ProTx-II. The authors included additional analogs that contained the disulfide-bond directing penicillamine residue at distinct positions of the peptides' C-terminus, in order to examine whether misfolded intermediates can be diminished and folding could be directed toward the native disulfide isomer. Although the desired 3-disulfide-bonded product was increased, sufficient evidence was found that additional 2-disulfide-bonded peptides were produced concomitantly, leaving open a wide scope for future studies. The folding pathway of a peptide derived from Amaranth seeds, the alpha-amylase inhibitor (AAI), was in the focus of the contribution by Juhász et al. Again, folding pathway studies form the basis of this research analyzing in more detail the oxidative folding of AAI, which was described to occur according to the hirudin-like pathway (Chang, 2011). The authors propose the folding of this peptide via a major folding intermediate possessing a vicinal disulfide bond that is assumed to be a kinetic trap, but may lead to the native fold of AAI due to a reduction of the number of other intermediate conformations. A third suitable candidate study was provided by Lee et al. who focused on neuropeptide relaxin-3 and its analogs in their study introducing grafted disulfide-stabilized scaffolds for agonist and antagonist production. The use of two such disulfide-stabilized scaffolds in the relaxin-3 B-chain led to an increase in structure formation, i.e., helicity, and stability. The differently grafted analogs, however, showed varying results concerning biological activity. While peptides containing the Veronica hederifolia Trypsin inhibitor (VhTI) related motif displayed poor binding and low potency at the relaxin family peptide-3 receptor, the apamin variants still exerted considerable activity and improved serum half-life. Such approaches and strategies will be even more important in the future, when we know significantly more about the individual folding pathways and how competitive distortions could be limited, which will facilitate the peptide design by using grafted scaffolds. Continuing the line and as a consequence of the aforementioned disulfide-containing antibodies, the study by Horx and Geyer addressed the current issue by focusing on the hinge-region of immunoglobulin G1 (IgG1) by comparing it to the designed hinge-type motif CHWECRGCRLVC provided by the same group. Such small motifs were described to represent ideal models for computational studies. Herein, well-tempered metadynamics was used to determine the available conformational space defined by the respective hinges revealing characteristic similarities of the designed and natural hinges e.g., concerning differences of the mobility of the individual strands of the hinge domains.

The two contributions from Wiedemann et al. and Ramanujam et al. are focused on the importance of NMR spectroscopy for the characterization and structure elucidation of cysteine-rich and multiple disulfide-bonded peptides and proteins. In a mini-review, Wiedemann et al. provide insights into NMR-based applications for the characterization of disulfide-containing biomolecules. In multiple examples, such as conotoxins and kinases, the authors illustrate the essential role of cysteine disulfide bonds for the stabilization of the respective structures and the resulting biological activities. Thereby, the use of high-resolving NMR techniques enable the accurate definition of the molecular peptide or protein structures. In this regard, the impact of the disulfide bonds was also highlighted because of the high abundance of cysteines and disulfide bonds within the proteome of different domains of life. Concerning the structure elucidation by NMR spectroscopy, one of the major limitations is the fact that the disulfide bond itself is not visible in the NMR spectrum, which makes it difficult to determine the correct disulfide-bond connectivity. To improve the elucidation of the disulfide bond connectivity within disulfide-rich peptides, Ramanujam et al. use residual dipolar couplings (RDCs). This approach enhances the resolution of the NMR structure and leads to a more accurate definition of the amino acid side chains. As a result, the geometry of the disulfide bonds and the connectivity between the cysteine residues can be resolved with high reliability.

Indeed, the structural analysis of disulfide-containing peptides and proteins is not only important for structure-activity-relationship studies in the drug development process of an existing lead compound, but also for theoretical approaches using computational tools which aim at a better understanding and improved prediction of e.g., folding pathways of multiple disulfide-bonded peptides in the context of isomer formation. Exploiting computers to study and predict the problems of protein/peptide folding, binding to receptors, protein engineering, and drug discovery has gained significant momentum in recent years with the incurrence of efficient data mining algorithms and advanced dynamical force fields which can model even the most complex of the systems, evolving structurally and dynamically with unabated accuracy (Huang et al., 2016; Rosenfeld et al., 2016; van Gunsteren et al., 2018; Mansbach et al., 2019). The accessibility to such highly refined and efficient computational resources, many of which are publicly accessible or *open source*, has paved the way for studying the structure-function relationships of biological systems over wide temporal and spatial scales. It has further enabled researchers to contribute and incorporate their ideas extensively toward additional development and customization of such reserves. Significant involvement of computational tools, either by themselves or in association with related experimental gears helps understand molecular-level phenomena in great detail. In particular, the creation of huge databases has certainly triggered the data-mining techniques to evolve as very efficient predictive and designing tools, expanding the paradigm of computation to an extent never conceived of before (Gómez-Bombarelli et al., 2018; Lee et al., 2018).

The present collection is exploring the chemical design and biomedical applications of disulfide rich peptides along with the challenges and rewards that are hidden therein. The role that disulfide crosslinks play in a peptide is very difficult to generalize, owing to the wide range of structural, functional and receptor diversity in such biomolecules. Nevertheless, for many peptides/proteins, a typical observation is that the cysteine residues and disulfide links play a crucial scaffolding role which indeed becomes decisive while binding to the corresponding receptor, sometimes even remotely from the actual binding site—these are the catalytic or allosteric disulfides (Cook and Hogg, 2013; Chen et al., 2014). Cysteine pairing, a major post-translational modification is also often accompanied by formation of disulfide bond isoforms, which poses a great challenge in their chemical synthesis and isolation. It is indeed challenging sometimes to figure out the correct disulfide pairing under experimental conditions, especially for peptides having multiple cysteine residues. X-ray, NMR, and Mass spectrometric techniques are the experimental tools of choice in elucidating such structures. In a recent advancement, for a three disulfide bond peptide, μ-PIIIA, multidimensional NMR, MS/MS and chemical synthetic strategies, like directed folding, proved convenient to successfully synthesize and characterize all the 15 disulfide bond isoforms (Heimer et al., 2018). Nonetheless, the inherent difficulty in the correct assignment of the disulfide bonds under experimental conditions may sometime result in the reporting of erroneous structures. In these situations, computation tools can really prove advantageous. A classic example is a work from the group of van Gunsteren, who used molecular dynamics method to prove that the NMR model structure of rice lipid transfer protein, LTP2, deposited in the protein data bank was both energetically unfavorable and structurally unstable and was a result of an experimental artifact (Allison et al., 2010).

Evidently, it is an era where along with experimental resources, computers must be exploited to the utmost, without any bias/prejudice, to reap the best results from all perspectives, both in academic and industrial setups. Finally, we hope that this issue of *Frontiers* will be of great interest to the Frontiers audience and will serve as an inspiration to advance research in the field of disulfide-rich peptides and proteins.

## Author Contributions

DI, FA, and DR have contributed in handling the manuscripts as Guest/Associate guest editors and have also written the editorial. All authors contributed to the article and approved the submitted version.

## Conflict of Interest

The authors declare that the research was conducted in the absence of any commercial or financial relationships that could be construed as a potential conflict of interest.
